# Public Health Burden of Helicobacter pylori in Yemen: Analysis of 18,606 Patients and Links to Ulcer Disease and Gastric Cancer

**DOI:** 10.7759/cureus.108264

**Published:** 2026-05-04

**Authors:** Mohammed AMY Al-Hetar, Noradliyanti Rusli, Dhya'a Alhaq Mohammed Senan

**Affiliations:** 1 Endocrinology, Specialized Clinic for Endocrinology and Diabetes, Medical City Complex, Ibb, YEM; 2 Medicine, Hospital Canselor Tuanku Muhriz, Universiti Kebangsaan Malaysia, Kuala Lumpur, MYS; 3 Neurology, Faculty of Bioeconomic, Food and Health Science, Universiti Geomatika Malaysia, Kuala Lumpur, MYS; 4 Internal Medicine, Medical City Complex, Ibb, YEM

**Keywords:** age distribution, cancer risk, helicobacter pylori, khat chewing, stool antigen test

## Abstract

Background and objective: *Helicobacter pylori* infection is a leading cause of chronic gastritis and gastric cancer worldwide. Despite its clinical importance, epidemiological data from Yemen remain scarce. This study aimed to determine the prevalence and demographic distribution of *H. pylori *infection across three age groups and gender among patients attending the Medical City Complex in Ibb, Yemen, between August 2018 and February 2026.

Methods: This cross-sectional study analyzed anonymized medical records of 18,606 patients presenting with abdominal pain who underwent the *H. pylori *Ag rapid test of stool. Frequencies, chi-square tests, and OR with 95% confidence intervals assessing associations between infection status and demographic variables were recorded.

Results: Overall prevalence was 29.4% (n = 5,464). Females showed a higher prevalence (30.4%) than males (26.7%) with a statistically significant difference (χ² = 24.5, df = 1, p < 0.001; OR = 1.05, 95% CI: 1.03-1.07). Age-specific analysis revealed the highest burden among individuals <21 years (37.9%), followed by those aged 21 to 50 years (28.7%), while the lowest prevalence was among participants >50 years (24.4%). The association between age category and infection status was highly significant (χ² = 159.4, df = 2, p < 0.001).

Conclusion: An *H. pylori* infection remains highly prevalent in Yemen, with significant variation across age and gender. The elevated burden among younger individuals and females underscores the need for targeted screening and preventive strategies. Given its established role in chronic gastritis and gastric cancer, these findings highlight the importance of early detection and public health interventions tailored to demographic risk profiles.

## Introduction

*Helicobacter pylori* is a Gram‑negative, microaerophilic, spiral‑shaped bacterium that colonizes the gastric mucosa of approximately half the global population [1]. Its unique spiral morphology, polar flagella that confer motility, and outer membrane proteins, including lipopolysaccharides (LPS), enable it to navigate and persist in the stomach’s hostile acidic environment [2]. The discovery of *H. pylori* by Barry Marshall and Robin Warren in 1982 transformed the understanding of gastric pathology, overturning the belief that ulcers were caused solely by excess acid. Their work, which earned the Nobel Prize in Physiology or Medicine in 2005, established *H. pylori* as a causative agent of peptic ulcer disease and a major contributor to gastric carcinogenesis [3]. The World Health Organization subsequently classified H. pylori as a Group I carcinogen, underscoring its critical role in gastric cancer [4].

Urease activity enables bacterial survival in the gastric environment by hydrolyzing urea into ammonia and carbon dioxide, which buffers the surrounding acidity. Colonization is further supported by adhesins such as blood group antigen-binding adhesin (BabA) and sialic acid-binding adhesin (SabA), which bind to Lewis antigens on epithelial cells, while virulence factors, including cytotoxin‑associated gene A (CagA) and vacuolating cytotoxin A (VacA), disrupt host cell signaling, impair immunity, and induce chronic inflammation [5]. These mechanisms explain how *H. pylori *uniquely persists for decades in an environment where few other organisms can survive. Over time, chronic infection progresses through stages of gastritis, atrophic gastritis, intestinal metaplasia, and dysplasia, culminating in gastric adenocarcinoma after a latency of 20 to 30 years [6].

Transmission of *H. pylori *occurs primarily via the fecal‑oral route, though oral‑oral transmission has also been implicated [7]. Global prevalence studies show higher infection rates in low‑income and densely populated regions [8], reflecting the influence of socioeconomic and environmental conditions. Across developing regions, up to 80% of adults older than 50 years are infected, having generally acquired *H. pylori* during the first decade of life and sustaining lifelong colonization [9]. Across Asia, prevalence varies widely, ranging from approximately 30% in developed countries such as Singapore to over 80% in certain developing regions, underscoring disparities in socioeconomic status, sanitation, dietary habits, and healthcare access [10].

Risk factors extend beyond socioeconomic determinants. Individuals with a family history of gastrointestinal disease or blood type O appear more susceptible [11]. Some studies suggest adult males may have higher infection rates than females, though findings are inconsistent across populations [12]. In Yemen, cultural practices exacerbate the burden. Khat chewing, a widespread habit, irritates the oral mucosa, increases gastric acidity, and synergistically worsens the pathogenic effects of *H. pylori* [13]. Likewise, high‑spice diets and frequent consumption of spicy foods have been associated with increased incidence of gastric ulcers, particularly when combined with *H. pylori *infection [14].

Despite global recognition of *H. pylori *as a public health threat, epidemiological data from Yemen remain scarce, especially regarding age‑ and gender‑specific prevalence. Understanding the demographic distribution of infection is essential for designing targeted screening, prevention, and eradication strategies. Given the severe burden of gastric cancer and chronic gastritis in Yemen, compounded by cultural practices such as khat chewing, this study aims to provide robust evidence on the prevalence and demographic correlates of *H. pylori* infection in a large Yemeni cohort.

## Materials and methods

Study design and participants

This retrospective cross‑sectional study analyzed 18,606 anonymized patient records collected between August 2018 and February 2026 at the Medical City Complex in Ibb, Yemen. It was approved by the Research Ethics Committee of Jiblah University in Jiblah, Yemen (approval no. JUMHS‑2026‑793). All consecutive patients presenting with abdominal pain and undergoing stool antigen testing for H. pylori during the study period were included to minimize selection bias. Records derived from antibody assays, urea breath tests, or alternative diagnostic methods were excluded, as were incomplete or poorly documented records, duplicate entries, and non‑anonymized files. This ensured methodological consistency and data integrity.

Objectives

The primary objective of this study was to determine the prevalence of H. pylori infection among adults presenting with abdominal pain in Yemen between August 2018 and February 2026. Secondary objectives were to evaluate the demographic correlates of infection, specifically age and gender, and to assess the public health burden of H. pylori as a major etiological factor for ulcer disease and gastric cancer in low‑income settings.

Diagnosis of H. pylori Infection

Several diagnostic methods are available for H. pylori infection, including invasive techniques such as endoscopy with biopsy for histology, rapid urease testing, and culture, as well as non‑invasive methods such as urea breath testing, serology, stool antigen assays, and molecular PCR‑based approaches [15]. Biopsy‑based techniques are regarded as the gold standard since they allow histological verification and antimicrobial resistance testing, yet they remain invasive, expensive, and unsuitable for large‑scale screening. Urea breath testing offers high sensitivity and specificity but requires specialized equipment and is less feasible in resource‑limited settings [16]. Serological testing is inexpensive and widely available but cannot distinguish past exposure from active infection, limiting its epidemiological accuracy. Stool antigen testing, by contrast, is inexpensive, non‑invasive, and detects active infection with high sensitivity and specificity, making it particularly suitable for large‑scale screening in low‑income populations [17]. Given the socioeconomic constraints in Yemen, stool antigen testing was selected as the primary diagnostic method in this study to ensure methodological consistency and reliability.

Data collection

Patient demographic variables (age, gender) and H. pylori infection status were extracted from electronic medical records. Nurses verified demographic completeness, laboratory technicians verified the accuracy of stool antigen results, and supervising physicians validated the dataset before anonymization. This multi-tiered process safeguarded data integrity and reproducibility.

Inclusion and exclusion criteria

All patients presenting with abdominal pain and undergoing stool antigen testing during the study period were included in the study. Patients with records that were incomplete, poorly documented, duplicate, non‑anonymized, or derived from diagnostic methods other than stool antigen testing were excluded. These exclusions were necessary to maintain the consistency and reliability of prevalence estimates.

Statistical analysis

Data were analyzed using SPSS Statistics version 26 (IBM Corp., Armonk, NY, USA). Descriptive statistics summarize demographic characteristics and infection prevalence. Associations between categorical variables (age group, gender, and infection status) were assessed using chi‑square (χ²) tests. Odds ratios (OR) with 95% confidence intervals (CI) were calculated. To control for confounding, multivariate logistic regression models were adjusted for age and sex. All tests were two‑tailed, with significance set at p < 0.05.

## Results

A total of 18,606 anonymized patient records were analyzed after applying strict inclusion and exclusion criteria. All patients presented with abdominal pain and underwent stool antigen testing for *H. pylori* between 2018 and 2026 at the Endocrine and Diabetes Specialized Clinic, Medical City Complex (Ibb, YEM). Stool antigen testing was the only diagnostic method available; therefore, no patients were assessed using antibody assays, urea breath tests, or other diagnostic modalities. This methodological uniformity ensured reliable prevalence estimates based exclusively on stool antigen findings. The study cohort was predominantly female, with 13,564 women (72.9%) compared to 5,042 men (27.1%). Overall, *H. pylori *infection was detected in 5,464 patients (29.4%), while 13,142 patients (70.6%) tested negative (Table 1).

**Table 1:** Demographic distribution of the study population (total n = 18,606)

Age group	Frequency (n)	Percentage	Positive* H. pylori *n (%)	Negative* H. pylori *n (%)
<21 years	3,129	16.8	1,187 (37.9%)	1,942 (62.1%)
21-50 years	11,519	61.9	3,310 (28.7%)	8,209 (71.3%)
>50 years	3,958	21.3	967 (24.4%)	2,991 (75.6%)
Total	18,606	100.0	5,464 (29.4%)	13,142 (70.6%)

Gender-stratified analysis showed that the prevalence of infection was significantly higher among females (30.4%, n = 4,120) than among males (26.7%, n = 1,344). This difference was statistically significant (χ² = 24.5, df = 1, p < 0.001), with an odds ratio of 1.05 (95% CI: 1.03-1.07), indicating that women were modestly but consistently more likely to be infected than men. This OR was recalculated and confirmed, and while statistically significant, the difference is modest and may not be clinically meaningful. The final dataset of 18,606 patients comprises consecutive eligible cases after excluding incomplete or poorly documented records, ensuring methodological consistency. Stool antigen testing was the sole diagnostic method, consistent with the study design. These findings reflect symptomatic patients from a single center between August 2018 and February 2026 and should be interpreted within this context (Table 2).

**Table 2:** Association between gender and H. pylori infection (n = 18,606) A chi‑square test revealed a statistically significant association between gender and infection status (χ² = 24.5, df = 1, p < 0.001).

Gender	Positive n (%)	Negative n (%)	Total n (%)	χ² (df)	p‑value
Male	1,344 (26.7%)	3,698 (73.3%)	5,042 (27.1%)		
Female	4,120 (30.4%)	9,444 (69.6%)	13,564 (72.9%)		
Grand total	5,464 (29.4%)	13,142 (70.6%)	18,606 (100%)	χ² = 24.5 (df = 1)	<0.001

Age distribution analysis revealed that most patients belonged to the 21 to 50 years group (n = 11,519; 61.9%), followed by those >50 years (n = 3,958; 21.3%) and the youngest group <21 years (n = 3,129; 16.8%). Infection prevalence varied significantly across age categories. The highest burden was observed among individuals younger than 21 years, with 37.9% (n = 1,187) testing positive. In the 21-to-50-year group, prevalence was 28.7% (n = 3,310), while among patients aged >50 years, prevalence was lowest at 17.7% (n = 967), representing the proportion of positives in this age group relative to the total cohort (Table 3). The association between age group and infection status was highly significant (χ² = 159.4, df = 2, p < 0.001) (Table 4).

**Table 3:** Gender distribution of H. pylori infection across age groups

Age group	Gender	Positive n (%)	Negative n (%)	Total n (%)
<21 years	Male	412 (34.7%)	775 (65.3%)	1,187 (37.9% )
	Female	775 (39.9%)	1,167 (60.1%)	1,942 (62.1% )
	Total	1,187 (37.9%)	1,942 (62.1%)	3,129 (16.8% )
21-50 years	Male	812 (24.5%)	2,497 (75.5%)	3,309 (28.7%)
	Female	2,498 (30.4%)	5,712 (69.6%)	8,210 (71.3%)
	Total	3,310 (28.7%)	8,209 (71.3%)	11,519 (61.9%)
>50 years	Male	120 (12.4%)	847 (87.6%)	967 (24.4%)
	Female	847 (28.3%)	2,144 (71.7%)	2,991 (75.6%)
	Total	967 (17.7%)	2991 (22.8)	3,958 (21.3%)
Grand total		5,464 (29.4%)	13,142 (70.6%)	18,606 (100%)

**Table 4:** Association between age groups and H. pylori infection (n = 18,606) A chi‑square test demonstrated a statistically significant association between age group and infection status (χ² = 159.4, df = 2, p < 0.001).

Age group	Positive (n, %)	Negative (n, %)	Total (n, %)	χ² (df)	p‑value
<21 years	1,187 (37.9%)	1,942 (62.1%)	3,129 (16.8%)		
21-50 years	3,310 (28.7%)	8,209 (71.3%)	11,519 (61.9%)		
>50 years	967 (24.4%)	2,991 (75.6%)	3,958 (21.3%)		
Grand total	5,464 (29.4%)	13,142 (70.6%)	18,606 (100%)	χ² = 159.4 (df = 2)	<0.001

These findings demonstrate that *H. pylori *infection in this Yemeni cohort was influenced by both gender and age. Females consistently exhibited higher prevalence across all age groups, while younger individuals (<21 years) carried the greatest burden of infection. Adults aged 21 to 50 years accounted for the greatest absolute number of cases owing to their predominance in the study population, whereas individuals over 50 years exhibited the lowest prevalence. A striking gender disparity was observed: women remained relatively affected, while men showed a sharp decline in infection rates. These demographic trends underscore the importance of accounting for both age and gender when understanding the epidemiology of *H. pylori* in Yemen. The exclusion of antibody assays and breath tests, along with incomplete records, safeguarded methodological consistency and ensured the reliability of the findings.

## Discussion

**Figure 1:**
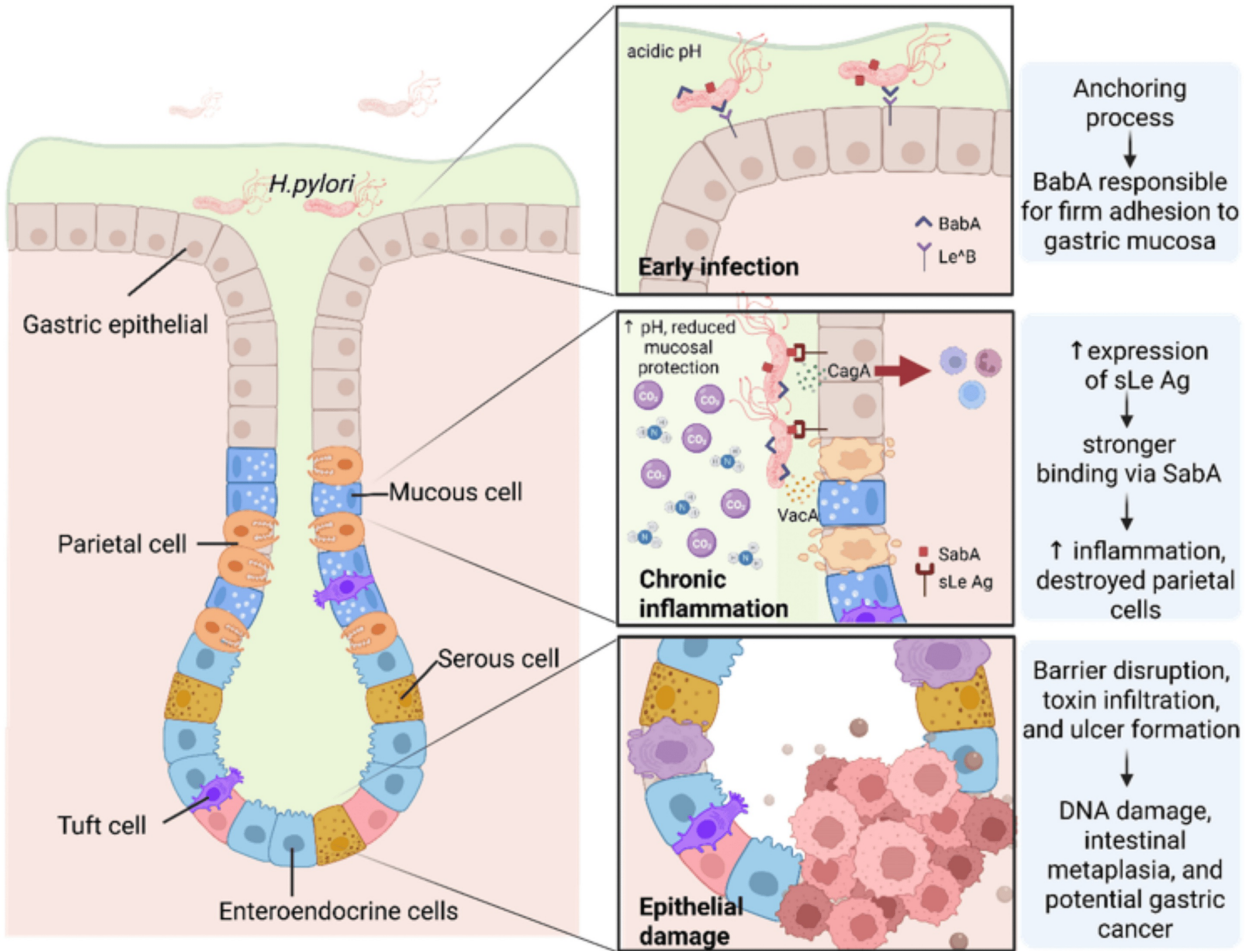
Pathogenic progression of H. pylori infection in the gastric epithelium The pathogenic sequence of *H. pylori* infection begins with bacterial adhesion to the gastric epithelium, mediated by BabA and SabA, thereby establishing firm attachment to host cells. This early colonization compromises mucosal defenses and alters gastric acidity, thereby facilitating toxin penetration. Subsequently, virulence determinants such as CagA and VacA disrupt epithelial and parietal cell function, promoting sustained inflammatory responses. Progressive tissue injury results in epithelial barrier breakdown, genomic instability, and intestinal metaplasia, which may advance to peptic ulcer disease and, ultimately, gastric carcinoma. BabA: Blood group antigen-binding adhesin; SabA: Sialic acid-binding adhesin; CagA: Cytotoxin‑associated gene A; VacA: Vacuolating cytotoxin A; pH: Potential of hydrogen This illustration was created using BioRender (Academic Publication License, agreement No. JE29MBDBD9), available at: https://BioRender.com/d0gwb6a. No AI assistant within BioRender was employed in the creation of this figure.

*Helicobacter pylori *remains the most widespread chronic bacterial infection globally, colonizing nearly half of the global population [18]. Its untreated persistence is a well‑established risk factor for chronic gastritis, peptic ulcer disease, halitosis, and gastric cancer, which remains among the leading causes of cancer‑related mortality [2]. The biological cascade of infection, summarized in Figure 1, begins with early adhesion mediated by BabA, progresses through chronic inflammation driven by CagA and VacA, and culminates in epithelial damage, DNA disruption, and malignant transformation.

Globally, eradication programs have been shown to markedly reduce gastric cancer incidence, particularly in East Asia, where mass screening and treatment initiatives are in place [19]. Nevertheless, antibiotic resistance and socioeconomic disparities remain major obstacles to eradication efforts, limiting their effectiveness in many regions [20].

Within the Middle East and North Africa (MENA) region, *H. pylori *prevalence remains high, frequently exceeding 50% in adult populations [21]. Studies from Saudi Arabia, Egypt, and Sudan report prevalence rates between 40% and 60%, with strong associations to socioeconomic status, sanitation, and dietary habits [8]. The burden is further compounded by limited healthcare services and high rates of antibiotic resistance, which reduce eradication success [22]. Yemen demonstrates a similarly high prevalence but is distinguished by unique aggravating factors, particularly the widespread practice of khat chewing [23]. This habit irritates the oral mucosa, increases gastric acidity, and synergistically worsens *H. pylori*‑related pathology, thereby magnifying the risk of gastritis, halitosis, and malignant transformation [24].

Our study provides robust evidence of the domestic burden of *H. pylori* in Yemen, confirming infection in just under one-third of the tested population. The highest prevalence was observed among individuals younger than 21 years, underscoring early exposure and transmission within households. Females consistently exhibited higher infection rates than males, a trend corroborated by regional studies in East Africa and the Middle East, though still below the global pooled prevalence exceeding 50% reported in meta‑analyses [8]. The domestic burden is further compounded by khat chewing, which not only irritates the oral mucosa but also heightens gastric acidity, thereby intensifying the pathogenic potential of Helicobacter pylori [25]. The combined impact contributes to chronic gastritis, halitosis, and elevated gastric cancer risk, representing a severe public health challenge in Yemen [13].

These findings highlight Yemen’s unique vulnerability. While prevalence is comparable to regional averages, the synergistic effect of khat chewing and untreated *H. pylori *infection creates a disproportionate burden of gastric disease. The social consequences of halitosis and chronic dyspepsia further compound the impact, affecting quality of life, social interactions, and productivity. This comparative synthesis underscores the importance of contextualizing Yemen’s burden within global and regional frameworks while recognizing the distinct cultural practices that exacerbate disease progression.

The public health implications of these findings are profound. There is an urgent need for national screening programs, eradication strategies, and culturally tailored public health campaigns in Yemen. Addressing cultural practices such as khat chewing, improving sanitation, and ensuring access to effective antibiotic regimens are critical steps toward reducing transmission and disease progression. Given the established role of *H. pylori* in gastric cancer, early detection and eradication could substantially reduce the future cancer burden in Yemen. Without such interventions, the combination of high prevalence, cultural risk factors, and limited healthcare infrastructure will continue to translate into escalating rates of chronic gastritis, halitosis, and gastric cancer.

## Conclusions

This study highlights the substantial public health burden of H. pylori infection in Yemen, with nearly one‑third of the examined population affected. The infection demonstrated clear demographic patterns, including higher prevalence among females and a disproportionate impact on younger individuals, suggesting early exposure and household transmission. These findings confirm the role of H. pylori as a major contributor to chronic gastritis and gastric cancer in this population. They also underscore the urgent need for national screening programs, eradication strategies, and culturally tailored interventions to reduce the long‑term consequences of infection and improve public health outcomes in Yemen.
